#  Association of Ambient and Household Air Pollution With Bone Mineral Content Among Adults in Peri-urban South India

**DOI:** 10.1001/jamanetworkopen.2019.18504

**Published:** 2020-01-03

**Authors:** Otavio T. Ranzani, Carles Milà, Bharati Kulkarni, Sanjay Kinra, Cathryn Tonne

**Affiliations:** 1 Barcelona Institute for Global Health, Universitat Pompeu Fabra, CIBER Epidemiología y Salud Pública, Barcelona, Spain; 2 National Institute of Nutrition, Indian Council of Medical Research, Hyderabad, India; 3 Department of Non-communicable Disease Epidemiology, London School of Hygiene and Tropical Medicine, London, United Kingdom

## Abstract

**Importance:**

Air pollution is a major threat to global health. Osteoporosis is responsible for a substantial burden of disease globally and is expected to increase in prevalence because of population aging. Few studies have investigated the association between air pollution and bone health, and their findings were inconclusive.

**Objective:**

To quantify the association between ambient and household air pollution and bone mass in a sample of the general population in peri-urban India.

**Design, Setting, and Participants:**

This was a population-based cross-sectional analysis of the Andhra Pradesh Children and Parents Study cohort, which recruited participants from 28 villages near Hyderabad, South India, during 2009 to 2012. Separate linear mixed models were fitted with nested random intercepts (household within villages) for each exposure-outcome pair and were sequentially adjusted for potential confounders. Data analysis was conducted between April 2019 and July 2019.

**Exposures:**

Annual mean ambient particulate matter air pollution less than 2.5 µm in aerodynamic diameter (PM_2.5_) and black carbon (BC) levels at the residence estimated by land-use regression and self-reported use of biomass cooking fuel.

**Main Outcomes and Measures:**

The primary outcome was bone mineral content (BMC) measured in grams, corrected by bone area at the lumbar spine and left hip, as measured by dual-energy x-ray absorptiometry. The secondary outcome was bone mineral density measured in grams per centimeters squared.

**Results:**

A total of 3717 participants were analyzed (mean [SD] age, 35.7 [14.0] years; 1711 [46.0%] women). The annual mean (SD) PM_2.5_ exposure was 32.8 (2.5) μg/m^3^, and the annual mean (SD) BC exposure was 2.5 (0.2) μg/m^3^; 57.8% of participants used biomass cooking fuels. In fully adjusted models, PM_2.5_ was associated with lower BMC in the spine (mean difference, −0.57 g per 3 μg/m^3^ increase in PM_2.5_; 95% CI, −1.06 to −0.07 g per 3 μg/m^3^ increase in PM_2.5_) and hip (mean difference, −0.13 g per 3 μg/m^3^ increase in PM_2.5_; 95% CI, −0.3 to 0.03 g per 3 μg/m^3^ increase in PM_2.5_). After confounder adjustment, exposure to PM_2.5_ was also associated with lower bone mineral density in the spine (mean difference, −0.011 g/cm^2^ per 3 μg/m^3^ increase in PM_2.5_; 95% CI, −0.021 to 0 g/cm^2^ per 3 μg/m^3^ increase in PM_2.5_) and hip (mean difference, −0.004 g/cm^2 ^per 3 μg/m^3^ increase in PM_2.5_; 95% CI, −0.008 to 0.001 g/cm^2^ per 3 μg/m^3^ increase in PM_2.5_). Exposure to BC was associated with lower BMC in the spine (mean difference, −1.13 g per 1 μg/m^3^ increase in BC; 95% CI, −2.81 to 0.54 g per 1 μg/m^3^ increase in BC) and hip (mean difference, −0.35 g per 1 μg/m^3^ increase in BC; 95% CI, −0.96 to 0.25 g per 1 μg/m^3^ increase in BC), although the confidence intervals were wider. There was no association between biomass fuel use and spine BMC (mean difference, 0.12 g; 95% CI, −0.45 to 0.68 g).

**Conclusions and Relevance:**

In a cross-sectional analysis of a population-based cohort, ambient air pollution was associated with lower BMC in a young adult population in a peri-urban area of South India.

## Key Points

**Question:**

Are ambient and household air pollution associated with bone mass among adults in a low- and middle-income country?

**Findings:**

In this population-based cross-sectional study of 3717 participants, ambient fine particulate matter air pollution was associated with low bone mineral content and bone mineral density. Household air pollution did not have a clear association with bone mass.

**Meaning:**

In a peri-urban population of India, ambient air pollution was associated with poorer bone health.

## Teaser

This population-based cross-sectional study analyzes the association between ambient and household air pollution and bone mass in a sample of the general adult population in peri-urban India.

## Introduction

Air pollution from outdoor and household sources is a public health concern and is responsible for a large proportion of morbidity and premature mortality.^1^ India, like many low- and middle-income countries, faces a large disease burden due to air pollution resulting from a combination of high levels of ambient air pollution, prevalent use of biomass cooking fuel, and population aging.^2,3,4^

Life spans have lengthened worldwide, and a larger number of people reaching ages beyond 65 years is expected to change population morbidity profiles, including an increase in the prevalence of osteoporosis.^4^ Osteoporosis increases the risk of subsequent osteoporotic fractures, reduced quality of life, and mortality.^5^ Osteoporosis, which is characterized by bones with low mass content and microarchitectural deterioration of bone tissue, is a final result of cumulative factors acting on skeletal health.^6,7^ These alterations are largely due to hormone-related and age-related bone losses or failure to achieve optimal peak bone mass during early adulthood.^6,7^

A large body of literature^8^ links ambient particulate matter air pollution (PM) with a wide range of noncommunicable diseases. Evidence evaluating the association between PM and skeletal health is limited.^9^ Some studies^10,11^ have observed associations between PM and lower bone mineral density (BMD), increased BMD loss, or increased risk of osteoporotic fracture. However, other studies have observed no association,^12^ and the overall evidence is mixed as to which age groups, sexes, and pollutants are most relevant.^13,14^ Available evidence is limited by small sample sizes and to high-income countries with relatively low levels of PM compared with the global range of exposure. In addition, to our knowledge, there is no study evaluating the association between bone mass and household air pollution (HAP) resulting from the use of biomass cooking fuels. The plausibility of an association between PM and worse bone health is supported by studies showing bone health deterioration associated with tobacco smoking.^15,16,17,18^ Indeed, PM promotes systemic inflammation and oxidative stress,^19^ which impair the bone remodeling process^7,20^ and indirectly alter bone hormonal homeostasis, such as via parathyroid hormone (PTH).^10^

This study contributes to the small and inconclusive body of evidence regarding the association between PM and bone health. Our objective was to quantify the association between ambient PM and HAP and bone health in a sample of the general population in a peri-urban area of India.

## Methods

### Study Population

We analyzed data from the third follow-up period (FU3; 2010-2012) of the Andhra Pradesh Children And Parents Study (APCAPS).^21^ The APCAPS is a large, prospective, intergenerational cohort study initiated through long-term follow-up of the Hyderabad Nutrition Trial (1987-1990).^21^ Participants in FU3 included parents and siblings of the index children who were born during the original trial (6944 participants).^21,22^ Participants resided in 28 villages outside the city of Hyderabad, India. Where data from FU3 were unavailable, we included data from the second follow-up period (FU2; 2009-2010). The FU2 data were collected only on the young adult index children (1446 participants). Ethics approval for APCAPS was granted by the Public Health Foundation of India, India, and the National Institute of Nutrition, Hyderabad, India. All participants provided written informed consent. This study follows the Strengthening the Reporting of Observational Studies in Epidemiology (STROBE) reporting guideline for cross-sectional studies.

### Data Collection

Data collection procedures during the 2 follow-up periods were comparable.^21^ During FU3, initial clinic visits occurred within villages, and participants were invited to attend a second clinic visit (transportation provided) at the National Institute of Nutrition (55% of FU3 participants attended second clinic visits), where the dual-energy x-ray absorptiometry (DXA) scans were conducted. During FU2, all data were collected at the National Institute of Nutrition.

### Outcome Assessment

We analyzed bone mineral content (BMC) in grams, bone area (BA) in centimeters squared, and bone mineral density (BMD) in grams per centimeters squared at left hip and lumbar spine (L1-L4) sites. Our primary outcome was BMC adjusted for BA, because this approach is more suitable to the evaluation of bone mineral determinants compared with BMD.^23,24^ Indeed, areal BMD is estimated by the ratio of BMC to BA and assumes a linear proportional association between BMC and BA, which is usually not a reasonable assumption.^23^ Our secondary outcome was BMD.

Bone area, BMC, and BMD were measured via DXA using a Discovery A scanner (Hologic) (84% of participants) and a 4500W scanner (Hologic) (16% of participants). The same scanners were used during FU2 and FU3 by a single, trained technician. Pregnant women were excluded from the scans.^25,26,27^ Standard Hologic software options were used to define regions of the body, and the same software version was used on both machines. Scans were visually inspected for artifacts; incomplete scans or those affected by major movement were excluded from analysis. For lumbar spine scans, pathological changes, such as osteoarthritis affecting 2 or more vertebrae, were excluded; if only 1 vertebra was affected, the scan was reanalyzed after the affected part was excluded.^25,26,27^ A spine phantom (spine phantom 14855, Hologic) was scanned every day to check for acceptable ranges. Agreement between repeated measures of BMD in a subset of participants was high (coefficient of variation, 0.7% for hip and 1.3% for lumbar spine), as was intrarater reliability (intraclass correlation coefficients, >0.995).^25,26,27^

### Exposure Assessment

Our exposures were fine particles, including PM less than 2.5 µm in aerodynamic diameter (PM_2.5_) and black carbon (BC). Annual mean exposures outdoors at residences were estimated using a land-use regression model developed for the local area within the Cardiovascular Health Effects of Air Pollution in Telangana, India Project,^28^ which builds on APCAPS by adding comprehensive assessment of exposure to air pollution in the APCAPS population. Ethics approval for the Cardiovascular Health Effects of Air Pollution in Telangana, India Project was granted by the Parc de Salut Mar, Spain, Public Health Foundation of India, and National Institute of Nutrition of India. Written informed consent was obtained from the participants. Model development and evaluation have been described elsewhere.^29^ Briefly, 24-hour gravimetric PM_2.5_ measurements were conducted in 23 locations in 2 different seasons. Measurements of BC were derived from optical attenuation of the mass collected on the filters using an OT21 Sootscan Optical Transmissometer (Magee Scientific). Factors associated with spatial variation in PM_2.5_ included remote sensing–derived tree cover, nighttime light intensity, normalized difference vegetation index, and longitude. Factors associated with variation in BC included the length of the ring road around Hyderabad, tree coverage, and distance to energy suppliers. The models explained 58% of the variance in PM_2.5_ and 78% of the variance in BC.^29^

We used self-reported main source of cooking fuel as an indicator of HAP. We derived a binary exposure by comparing electricity and liquefied petroleum gas with biomass fuels, kerosene, and oil.

### Covariate Data

Fat and lean body mass were calculated according to the whole-body DXA scans. Other covariate data were collected via standardized questionnaires, which included demographic characteristics, socioeconomic position (education, occupation, and standard of living index), health behaviors (smoking, diet, and physical activity), and household characteristics.^21,30^ Dietary intake over the past year was estimated with a validated semiquantitative food-frequency questionnaire.^31^ Physical activity data were collected using a validated questionnaire described elsewhere.^32^ In brief, participants were asked to recall the frequency and time spent in activities during the past week according to the following domains: work, travel, leisure (sports, games, and exercise), household, and sedentary and sleep. We calculated weight-bearing physical activity (hours per week) by adding the time spent in activities involving running, walking, standing, and carrying weights.^32^

### Statistical Analysis

Data analysis was conducted between April 2019 and July 2019. For each participant, we used outcome DXA data from FU3 when available; otherwise, we used data from FU2 (3812 participants from FU3 + 470 participants from FU2 = 4282 participants) because of the short time elapsed between the 2 follow-up periods and the compatibility of the measurements. We selected covariates for each participant using covariate measurements in the same follow-up period of the outcome. We excluded 377 participants younger than 18 years, resulting in 3905 participants as the target population. We excluded 130 participants missing ambient air pollution exposure data because their households could not be geocoded accurately, 51 participants because of missing fuel use, and 7 participants with missing covariate data. The missingness pattern is shown in eAppendix 1 in the Supplement. Because of the small proportion of missing data, we based our analysis on participants with complete data (3717 of 3905, or 95% of the target population).^33^

We estimated associations between annual ambient PM_2.5_ and BC and household cooking fuel and hip and spine BMC using separate linear mixed models, accounting for the hierarchical structure of our data (individuals clustered within households and households within villages) with nested random intercepts for each exposure-outcome pair. We defined our set of potential confounding factors according to a directed acyclic graph (eFigure 1 in the Supplement), using previous knowledge and reported associations in APCAPS population.^26,27^ We sequentially adjusted for confounders. Model 1 was adjusted for BA (natural cubic spline with 3 *df*), a DXA machine indicator, sex, age (second-degree polynomial), and a sex-by-age interaction. Model 2 was further adjusted for percentage lean and percentage fat body mass. Model 3 added to model 2 log-transformed intake of fruit, vegetables, and calcium; weight-bearing physical activity; smoking status; and household cooking fuel (in ambient models only). Finally, model 4 (main model) was also adjusted for socioeconomic confounders, including occupation, education, and standard of living index. In cooking fuel models, 2 separate models with and without an exposure-sex interaction were fit because of the sex-associated differences in cooking time, which may be associated with HAP exposure.^34^ We used inverse probability weighting (IPW)^35^ to account for differences between the 3717 participants included in analyses and the adult population of 5989 participants included in APCAPS, which were representative of the general population.^22,30^ More details about IPW are given in eAppendix 2 in the Supplement. We evaluated whether PM_2.5_ and BC had a nonlinear association with BMC and BMD using thin-plate splines in generalized additive mixed models.

As sensitivity analyses, we fitted models without IPW; used a within-between village model specification^36^ in ambient models to differentiate the between-village and within-village associations, motivated by potential confounding at the village level in similar studies of the APCAPS population^22,30^; analyzed BMD as an outcome; fitted our main model (model 4) with both PM_2.5_ and BC exposures to compare the degree of association between the 2 particle exposure metrics; and fitted our main analysis on participants aged 40 years and older, to account for the main period of bone loss. Analyses were performed in R statistical software version 3.5.3 (R Project for Statistical Computing)^37^ using the set of tidyverse packages^38^ for data management and lme4 for linear mixed model estimation.^39^

## Results

### Study Population

Table 1 provides the general characteristics of the 3717 participants analyzed. Their mean (SD) age was 35.7 (14.0) years, and 1711 (46.0%) were women. Participants’ age distribution was bimodal (eFigure 2 in the Supplement), reflecting the index children and siblings and their parents. Nearly one-half of participants (1802 [48.5%]) did not have formal education, and 1742 (46.9%) worked in unskilled manual jobs. Regarding health behaviors, 944 participants (25.4%) were currently using tobacco.

**Table 1.** General Characteristics, Outcome, and Exposure Description of the Study Population

Characteristic	Participants, No. (%)		
	All (N = 3717)	Men (n = 2006)	Women (n = 1711)
Age, arithmetic mean (SD), y	35.7 (14.0)	34.7 (15.5)	36.8 (12.1)
Education			
No formal education	1802 (48.5)	682 (34.0)	1120 (65.5)
Primary school	471 (12.7)	314 (15.7)	157 (9.2)
Secondary school	1198 (32.2)	832 (41.5)	366 (21.4)
Superior studies	246 (6.6)	178 (8.9)	68 (4.0)
Occupation			
Unskilled manual	1742 (46.9)	804 (40.1)	938 (54.8)
Skilled manual	783 (21.1)	609 (30.4)	174 (10.2)
Nonmanual	158 (4.3)	128 (6.4)	30 (1.8)
Unemployed	1034 (27.8)	465 (23.2)	569 (33.3)
Standard of living index, arithmetic mean (SD)	29.1 (8.7)	29.9 (8.7)	28.2 (8.5)
Current tobacco use^a^	944 (25.4)	700 (34.9)	244 (14.3)
Weight-bearing physical activity, arithmetic mean (SD), h/wk	4.2 (3.2)	4.8 (3.0)	3.5 (3.3)
Height, arithmetic mean (SD), cm	158.6 (9.1)	164.6 (6.8)	151.5 (5.9)
Fat body mass, arithmetic mean (SD), %	12.7 (5.9)	10.3 (5.0)	15.5 (5.7)
Lean body mass, arithmetic mean (SD), %	38.1 (8.2)	43.4 (6.6)	31.9 (4.9)
Calcium intake, geometric mean (geometric SD), mg/d	425.7 (1.7)	466.3 (1.7)	382.5 (1.7)
Fruit and vegetable intake, geometric mean (geometric SD), g/d	203.6 (1.9)	228 (1.9)	178.3 (1.8)
Hip bone mineral content, arithmetic mean (SD), g	29.6 (7.0)	34.1 (5.6)	24.2 (4.1)
Hip bone area, arithmetic mean (SD), cm^2^	33.2 (5.0)	36.7 (3.7)	29.2 (2.7)
Hip bone mineral density, arithmetic mean (SD), g/cm^2^	0.88 (0.13)	0.93 (0.12)	0.83 (0.11)
Lumbar spine bone mineral content, arithmetic mean (SD), g	48.3 (12.4)	54.3 (12)	41.4 (8.9)
Lumbar spine bone area, arithmetic mean (SD), cm^2^	53.2 (6.6)	57.3 (5.2)	48.4 (4.5)
Lumbar spine bone mineral density, arithmetic mean (SD), g/cm^2^	0.9 (0.15)	0.94 (0.16)	0.85 (0.14)
Ambient particulate air pollution <2.5 µm in aerodynamic diameter, arithmetic mean (SD), μg/m^3^	32.8 (2.5)	32.8 (2.5)	32.8 (2.6)
Ambient black carbon, arithmetic mean (SD), μg/m^3^	2.5 (0.2)	2.5 (0.2)	2.5 (0.2)
Primary cooking fuel biomass	2148 (57.8)	1107 (55.2)	1041 (60.8)

^a^ Current tobacco use includes smoking, chewing, or snuffing tobacco in the last 6 months.

Men and women had the expected age-related bone patterns for BMC and BMD (eFigure 2 in the Supplement). The association between BMC and BA was nonlinear for both hip and spine sites (eFigure 3 in the Supplement).

**Figure 1.**
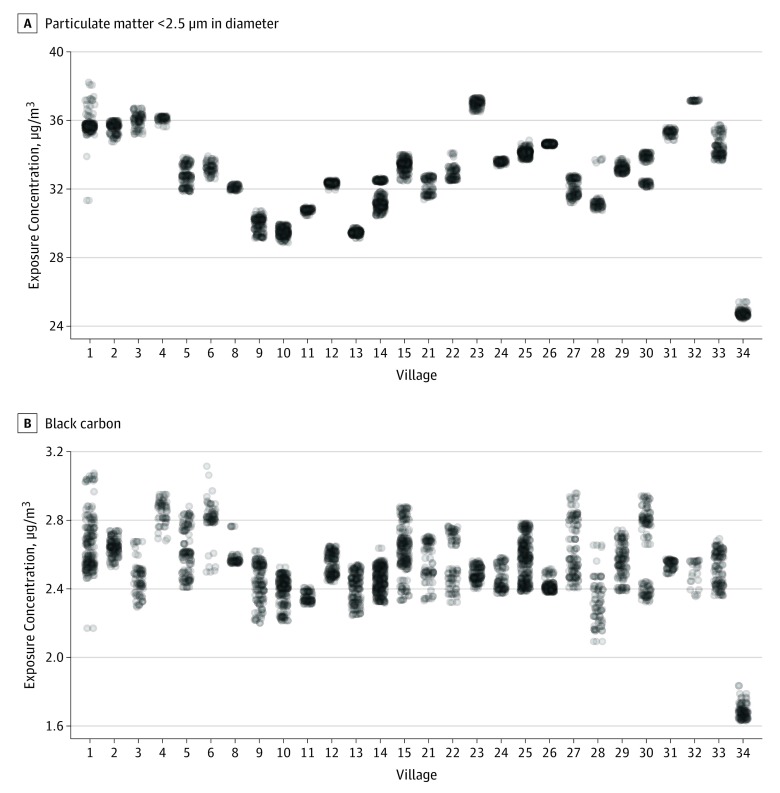
Annual Ambient Particulate Matter Exposure Distribution by Study Village Exposure concentrations of ambient particulate matter air pollution less than 2.5 µm in aerodynamic diameter (A) and black carbon (B) are shown by study village. Numbers on the x-axes represent village identification numbers and do not follow a numeric sequence or denote a geographical location.

Annual mean (SD) exposure to ambient PM_2.5_ was 32.8 (2.5) μg/m^3^ and that to ambient BC was 2.5 (0.2) μg/m^3^. The exposure range to both ambient PM_2.5_ and BC varied within and between the 28 villages (Figure 1). The correlation between PM_2.5_ and BC was moderate (Pearson *R* = 0.65). Overall, 57.8% of the population reported using biomass products as the primary cooking fuel (biomass fuel, 2097 participants [56.4%]; kerosene, 46 participants [1.3%]; and oil, 5 participants [0.1%]); 37 participants (1.0%) reported using electricity, and 1531 participants (41.2%) reported using liquefied petroleum gas (Table 1).

### Associations Between Ambient Air Pollution and BMC

Overall, there was a negative association between ambient PM_2.5_ and BMC at the hip and spine, with point estimates larger for the spine compared with the hip (Table 2). In the main model (model 4), PM_2.5_ was associated with lower BMC in the spine (mean difference, −0.57 g per 3 μg/m^3^ increase in PM_2.5_; 95% CI, −1.06 to −0.07 g per 3 μg/m^3^ increase in PM_2.5_) and hip (mean difference, −0.13 g per 3 μg/m^3^ increase in PM_2.5_; 95% CI, −0.3 to 0.03 per 3 μg/m^3^ increase in PM_2.5_). Black carbon was associated with lower BMC in the spine (mean difference, −1.13 g per 1 μg/m^3^ increase in PM_2.5_; 95% CI, −2.81 to 0.54 g per 1 μg/m^3^ increase in PM_2.5_) and hip (mean difference, −0.35 g per 1 μg/m^3^ increase in PM_2.5_; 95% CI, −0.96 to 0.25 g per 1 μg/m^3^ increase in PM_2.5_), although the confidence intervals were wider (Table 2).

**Table 2.** Association Between Annual Ambient Particulate Matter Air Pollution and Bone Mineral Content Corrected by Bone Area at the Hip and Lumbar Spine Sites^a^

Site, Model	Bone Mineral Content, Mean Difference (95% CI), g	
	Per 3 μg/m^3^ Increase in PM_2.5_	Per 1 μg/m^3^ Increase in BC
Hip		
Model 1^b^	−0.14 (−0.39 to 0.10)	−0.80 (−1.59 to −0.02)
Model 2^c^	−0.15 (−0.32 to 0.02)	−0.39 (−1.01 to 0.23)
Model 3^d^	−0.13 (−0.29 to 0.03)	−0.36 (−0.96 to 0.25)
Model 4^e^	−0.13 (−0.30 to 0.03)	−0.35 (−0.96 to 0.25)
Lumbar spine		
Model 1^b^	−0.62 (−1.13 to −0.10)	−1.22 (−2.95 to 0.51)
Model 2^c^	−0.62 (−1.12 to −0.11)	−1.18 (−2.89 to 0.53)
Model 3^d^	−0.59 (−1.09 to −0.09)	−1.17 (−2.86 to 0.53)
Model 4^e^	−0.57 (−1.06 to −0.07)	−1.13 (−2.81 to 0.54)

Abbreviations: BC, black carbon; PM_2.5_, ambient particulate matter air pollution less than 2.5 µm in aerodynamic diameter.

^a^ Associations were estimated using mixed effects linear models with nested random intercepts (household within village) using inverse-probability weighting.

^b^ Model 1 was adjusted for bone area (natural cubic spline with 3 *df*), a dual-energy x-ray absorptiometry machine indicator, sex, age (second-degree polynomial), and a sex-by-age interaction.

^c^ Model 2 included model 1 and was further adjusted for percentage lean and percentage fat body mass.

^d^ Model 3 added to model 2 log-transformed intake of fruit, vegetables, and calcium; weight-bearing physical activity; smoking status; and household cooking fuel.

^e^ Model 4 (main model) was also adjusted for socioeconomic confounders, including occupation, education, and standard of living index.

The sensitivity analysis for BMC without using IPW showed similar results (eTable 1 in the Supplement). The within-between village model specification also showed similar results, except for PM_2.5_ and hip, when the point estimate of the within-village association was more negative than the between-village association, but with wider confidence intervals (eFigure 4 in the Supplement).

When considering BMD as an outcome (eTable 2 in the Supplement), we observed similar findings as for BMC corrected by BA, such as a negative association between PM_2.5_ and BMD for spine (mean difference, −0.011 g/cm^2^ per 3 μg/m^3^ increase in PM_2.5_; 95% CI, −0.021 to 0 g/cm^2^ per 3 μg/m^3^ increase in PM_2.5_, model 4) and hip (mean difference, −0.004 g/cm^2 ^per 3 μg/m^3^ increase in PM_2.5_; 95% CI, −0.008 to 0.001 g/cm^2^ per 3 μg/m^3^ increase in PM_2.5_, model 4).

In multipollutant models, the associations had comparable point estimates for PM_2.5_, with less precision, and shifted to the null for BC (eTable 3 in the Supplement). Among those aged 40 years and older (Table 3), we observed the same pattern of association for PM_2.5_ and BC, with higher magnitude and precision of point estimate for PM_2.5_ and BMC (hip, mean difference, −0.37 g per 3 μg/m^3^ increase in PM_2.5_ [95% CI, −0.63 to −0.11 g per 3 μg/m^3^ increase in PM_2.5_]; spine, mean difference, −0.86 g per 3 μg/m^3^ increase in PM_2.5_ [95% CI, −1.66 to −0.06 g per 3 μg/m^3^ increase in PM_2.5_] model 4). For BC, BMC in the hip decreased by −0.69 g per 1 μg/m^3^ increase in BC (95% CI, −1.64 to 0.27 g per 1 μg/m^3^ increase in BC), and BMC in the spine decreased by −1.20 g per 1 μg/m^3^ increase in BC (95 % CI, −3.91 to 1.51 g per 1 μg/m^3^ increase in BC) (eTable 4 and eTable 5 in the Supplement). There was no evidence of departure from linearity between PM_2.5_ and BC and between BMC and BMD.

**Table 3.** Adjusted Associations Between Annual Ambient Particulate Matter Air Pollution and Bone Mineral Mass at the Hip And Lumbar Spine Sites Among Those Aged 40 Years or Older^a^

Variable	Bone Mineral Mass, Mean Difference (95% CI)	
	Per 3 μg/m^3^ Increase in PM_2.5_	Per 1 μg/m^3^ Increase in BC
Bone mineral content, g		
Left hip^b^	−0.37 (−0.63 to −0.11)	−0.69 (−1.64 to 0.27)
Lumbar spine^b^	−0.86 (−1.66 to −0.06)	−1.20 (−3.91 to 1.51)
Bone mineral density, g/cm^2^		
Left hip^c^	−0.010 (−0.016 to −0.003)	−0.019 (−0.044 to 0.007)
Lumbar spine^c^	−0.018 (−0.038 to 0.001)	−0.007 (−0.066 to 0.052)

Abbreviations: BC, black carbon; PM_2.5_, ambient particulate matter air pollution less than 2.5 µm in aerodynamic diameter.

^a^ Associations were estimated using mixed effects linear models with nested random intercepts (household within village) using inverse-probability weighting.

^b^ Model 4 (main model) was adjusted for natural spline (bone area); a sex-by-age interaction; dual-energy x-ray absorptiometry machine indicator; height; percentage fat body mass; percentage lean body mass; weight-bearing physical activity; log-transformed intake of fruit, vegetables, and calcium; current tobacco use; primary cooking fuel; occupation; education; and socioeconomic confounders, including occupation, education, and standard of living index.

^c^ Model 4 (main model) was adjusted for sex-by-age interaction; dual-energy x-ray absorptiometry machine indicator; height; percentage fat body mass; percentage lean body mass; weight-bearing physical activity; log-transformed intake of fruit, vegetables, and calcium; current tobacco use; primary cooking fuel; occupation; education; and socioeconomic confounders, including occupation, education, and standard of living index.

### Associations Between HAP and BMC

**Figure 2.**
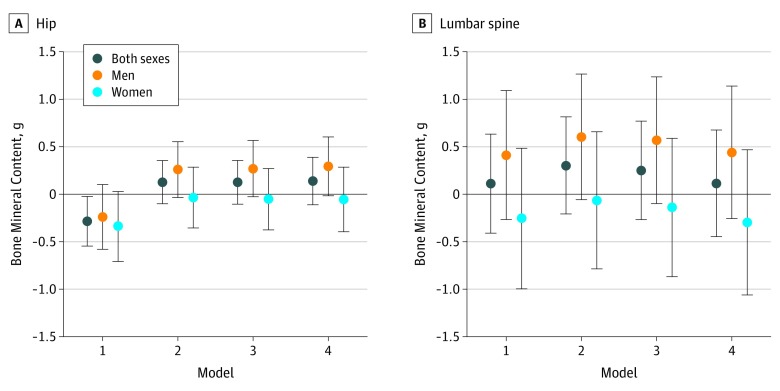
Association Between Biomass Fuel and Hip or Lumbar Spine Bone Mineral Content Corrected by Bone Area in Total Population and With Exposure-Sex Interaction Bone mineral content in the hip (A) and lumbar spine (B) is shown according to model. Dots denote mean differences, and vertical lines denote 95% CIs. Mixed effects linear models were run with nested random intercepts (household within village) using inverse-probability weighting. Two different models with and without exposure-sex interactions were run. Model 1 was adjusted for bone area (natural cubic spline with 3 *df*), a dual-energy x-ray absorptiometry machine indicator, sex, age (second-degree polynomial), and a sex-by-age interaction. Model 2 was further adjusted for percentage lean and percentage fat body mass. Model 3 added to model 2 log-transformed intake of fruit, vegetables, and calcium; weight-bearing physical activity; smoking status; and household cooking fuel (in ambient models only). Model 4 (main model) was also adjusted for socioeconomic confounders, including occupation, education, and standard of living index.

The association between cooking with biomass fuel and BMC was negative for the hip in all participants and for both men and women when adjusted for the minimal set of confounders. There also was no association between biomass fuel use and spine BMC. However, after further adjustment, the direction of the point estimates was reversed in all participants (mean difference, 0.12 g; 95% CI, −0.45 to 0.68 g, model 4) and among men compared with the minimal adjustment (Figure 2). For the spine, the associations were positive among all participants and among men, but negative among women. Compared with the associations for the hip, the change in point estimates after adjusting for confounding was less pronounced, and there was greater uncertainty in the estimates (Figure 2). The same pattern was observed for the association between biomass fuel and BMD, and among those aged 40 years and older (eFigure 5, eFigure 6, and eFigure 7 in the Supplement).

## Discussion

In this population-based cross-sectional study in a peri-urban area of South India, we observed that ambient air pollution, particularly ambient PM_2.5_, was associated with low bone mass. We did not observe a clear association between use of biomass as main cooking fuel and bone mass.

Several potential mechanisms may play a role in the association between PM and skeletal health. Inhalation of combustion particles may lead to increased bone mineral loss via systemic oxidative stress or inflammation,^19^ both of which are established mechanisms for bone demineralization and osteoporosis.^7,20^ Evidence from animal studies suggests a possible role of benzo(a)pyrene, a combustion by-product found in biomass fuel smoke,^40^ on bone resorption.^18^ There is also evidence of an association between PM and markers of bone turnover in children^41^ and between long-term exposure to solid fuel combustion in premenopausal women in India and the receptor activator of nuclear factor-kappa ligand 1–osteoprotegerin pathway, which regulates bone metabolism.^42^ We found that the association between PM_2.5_ and low bone mass was greater for the lumbar spine, which is mainly composed of trabecular bone (~80%),^7^ than for the hip, which has a higher proportion of cortical bone. This finding could be explained by higher sensitivity of the trabecular bone compared with cortical bone to the underlying oxidative stress generated by PM.^43^ These pathways should be further investigated, both in experimental and clinical or epidemiological settings.

Other indirect pathways, such as through vitamin D and PTH, are also implicated in the harmful associations of air pollution with bone modeling and remodeling homeostasis. First, ambient air pollution (PM and ozone) presents a physical barrier to solar ultraviolet B radiation,^44^ thereby contributing to lower cutaneous production of vitamin D.^12,45,46^ Low levels of vitamin D are widespread in India, reflecting a combination of factors, including diets low in calcium and vitamin D, skin pigmentation, and traditional clothing.^47,48^ Second, a recent study^10^ in the United States found an association between 1-year residential concentration of ambient PM_2.5_ and BC and lower serum PTH levels. Thus, the effect of air pollution on the vitamin D–PTH axis, a major contributor to skeletal health maintenance, might be similar to that observed for tobacco exposure, which has direct and indirect harmful effects on bone mass.^49^

Direct comparisons between our estimates and previous studies is challenging because of the small number of studies on this topic, diverse population characteristics, and differences in air pollution and outcome measurement methods. A study^11^ in Oslo, Norway, observed a negative association between PM_2.5_ (mean PM_2.5_, 12.4 μg/m^3^) and hip BMD in 518 older men, with an estimate of −0.009 g/cm^2^, compared with −0.004 g/cm^2^ in our study (point-estimate converted from milligrams per centimeters squared per 10 μg/m^3^ to grams per centimeter squared per 3 μg/m^3^ increase). The larger estimate observed in Oslo might be due to increased susceptibility to air pollution exposure associated with age in the Norwegian study, as we observed among those aged 40 years and older in our study.

Household air pollution due to inefficient fuel combustion is responsible for a large part of the burden of disease attributed to air pollution worldwide. Personal exposure and kitchen concentrations of PM can be extremely high when cooking.^34,50,51^ The association between primary cooking fuel and bone mass was not clear in our study, which may reflect exposure measurement error based on self-reported cooking fuel use. Fuel stacking is prevalent in this population,^34^ and our self-reported exposure does not capture the complexity of actual cooking fuel use. However, we did observe that men and women had different associations between biomass cooking fuel and bone mass, which might be the result of the considerably longer time spent by women cooking and in the kitchen compared with men in this population.^30,52^ Our previous work^53^ has shown that women have higher measured personal exposure to particles, and this exposure is largely driven by cooking with biomass fuel.

The number of osteoporotic fractures is expected to increase considerably over the next decades, particularly for non-Western populations.^7^ For example, projections indicate that 51% of hip fractures will occur in Asia by 2050,^54^ a region experiencing rapid population aging and urbanization. Air pollution could play an important role in mediating the association between urbanity and skeletal health. Evidence suggests that rural populations have lower risk of osteoporotic fractures and better skeletal health compared with urban populations, a difference that cannot be attributed only to differences in lifestyle and health behaviors.^55^ A systematic review^56^ of 15 articles reported that the urban-rural difference was observed only in high-income countries and speculated that lower air pollution levels in rural areas of high-income countries, together with other environmental factors, could be one explanation for the difference. Our study adds to this evidence by providing findings from a peri-urban area experiencing rapid urbanization, which entails increasing ambient air pollution over time alongside decreasing HAP from improved access to clean cooking fuel.

### Strengths and Limitations

Strengths of this study include the use of a population-based cohort, a relatively large sample size compared with other studies on this topic, and locally derived ambient air pollution models.^21,29^ In addition, to our knowledge, this is the first study in a setting with high levels of PM that bridge the lower levels of ambient particles and environmental tobacco smoke discussed in previous literature. We also used BMC corrected by BA, which does not assume a linear proportion between BMC and BA as areal BMD and, thus, is more suitable for epidemiological studies looking for determinants of skeletal health.^23,24^ Nevertheless, this study has important limitations that should be considered. First, this is a cross-sectional study; therefore, we could not evaluate the association between air pollution and bone loss over time or the incidence of osteoporotic fractures. A longitudinal design might have increased our statistical power and precision of the estimates.^10^ Second, we relied on self-reported primary fuel use measured by questionnaires, which likely resulted in exposure misclassification that may have biased our estimates toward the null. We did not have data on the lifetime history of household biomass fuel use. More comprehensive data on where households are in the transition from biomass to clean fuels (eg, liquefied petroleum gas), which is an important transition that is under way in India,^57^ would have improved our ability to estimate the association of HAP and skeletal health. We cannot rule out the potential for residual confounding, from unmeasured confounders or through measurement error of variables correlated with biomass, such as physical activity. The majority of physical activity in this population is occupation related, with higher levels of activity in more rural villages, which also rely more on biomass cooking fuel. More detailed, objectively measured, physical activity data could have reduced the association of this important confounder with the HAP findings. Fourth, we used IPW to account for selection bias by creating a pseudopopulation,^30,35^ but we cannot rule out potential residual selection bias due to small differences between the pseudopopulation and the target population. Fifth, we did not have data on heavy metals, such as lead and cadmium, that affect bone metabolism and are associated with air pollution.^11,13,58^ Sixth, vitamin D was measured in only a subsample of participants during FU2, and we did not have data on PTH. Although we could not explore their potential role as mediators, the influence of vitamin D may be limited. This is supported by a previous study^26^ in the FU2 subsample, which showed no clear association between vitamin D and BMD, even though vitamin D levels were low (<20 ng/mL for approximately 60% of participants).

## Conclusions

In a young adult population in a peri-urban area of South India, we observed an association between ambient PM_2.5_ and BC and low bone mass. The association between the use of biomass fuels as the main source of cooking fuel and low bone mass was not clear. Further studies in areas with high levels of air pollution from outdoor and household sources, investigating bone mass but also the incidence of osteoporotic fractures, are needed to better quantify the current and future burden of air pollution on bone health.

## Notes

Supplement. **eAppendix 1.** Missing Data Description and Missingness Pattern **eFigure 1.** Directed Acyclic Graph (DAG) Used to Select Confounding Factors **eAppendix 2.** Inverse Probability Weighting to Account for Potential Selection Bias **eFigure 2.** Associations Between Age and Bone Mineral Content (BMC), Bone Area (BA), and Bone Mineral Density (BMD) by Sex **eFigure 3.** Associations Between Bone Mineral Content (BMC) and Bone Area (BA) **eTable 1.** Mean Difference (95% CI) in Bone Mineral Content per Interquartile Range Increase in Annual Ambient Particulate Air Pollution, Without Inverse-Probability Correction (IPW) **eFigure 4.** Association Between Long-Term Ambient Particulate Air Pollution and Hip/Lumbar Spine Bone Mineral Content Corrected by Bone Area With vs Without Between-Within Exposure Specification **eTable 2.** Mean Difference (95% CI) in Bone Mineral Density (BMD) per Interquartile Range Increase in Annual Ambient Particulate Air Pollution **eTable 3.** Single and Multi-Pollutant Models for the Mean Difference (95% CI) in Bone Mineral Content and Bone Mineral Density per Interquartile Range Increase in Annual Ambient Particulate Air Pollution **eTable 4.** Association Between Annual Ambient Particulate Air Pollution and Bone Mineral Content at the Hip and Lumbar Spine Sites Among Those Aged ≥40 Years **eTable 5.** Association Between Annual Ambient Particulate Air Pollution and Bone Mineral Density at the Hip and Lumbar Spine Sites Among Those Aged ≥40 Years **eFigure 5.** Association Between Biomass Fuel and Hip/Lumbar Spine Bone Mineral Density (BMD) in All Population vs Exposure-Sex Interaction **eFigure 6.** Association Between Biomass Fuel and Hip/Lumbar Spine Bone Mineral Content Corrected by Bone Area in All Population vs Exposure-Sex Interaction (Among Those Aged ≥40 Years) **eFigure 7.** Association Between Biomass Fuel and Hip/Lumbar Spine Bone Mineral Density (BMD) in All Population vs Exposure-Sex Interaction (Among Those Aged ≥40 Years) **eReferences.**
