# Supplementary material for: Association of Ambient and Household Air Pollution With Bone Mineral Content Among Adults in Peri-urban South India
Source: JAMA Netw Open. 2020 Jan 3;3(1):e1918504. doi: 10.1001/jamanetworkopen.2019.18504 (PMC6991311; doi:10.1001/jamanetworkopen.2019.18504)
Supplement: Supplement. — eAppendix 1. Missing Data Description and Missingness Pattern eFigure 1. Directed Acyclic Graph (DAG) Used to Select Confounding Factors eAppendix 2. Inverse Probability Weighting to Account for Potential Selection Bias eFigure 2. Associations Between Age and Bone Mineral Content (BMC), Bone Area (BA), and Bone Mineral Density (BMD) by Sex eFigure 3. Associations Between Bone Mineral Content (BMC) and Bone Area (BA) eTable 1. Mean Difference (95% CI) in Bone Mineral Content per Interquartile Range Increase in Annual Ambient Particulate Air Pollution, Without Inverse-Probability Correction (IPW) eFigure 4. Association Between Long-Term Ambient Particulate Air Pollution and Hip/Lumbar Spine Bone Mineral Content Corrected by Bone Area With vs Without Between-Within Exposure Specification eTable 2. Mean Difference (95% CI) in Bone Mineral Density (BMD) per Interquartile Range Increase in Annual Ambient Particulate Air Pollution eTable 3. Single and Multi-Pollutant Models for the Mean Difference (95% CI) in Bone Mineral Content and Bone Mineral Density per Interquartile Range Increase in Annual Ambient Particulate Air Pollution eTable 4. Association Between Annual Ambient Particulate Air Pollution and Bone Mineral Content at the Hip and Lumbar Spine Sites Among Those Aged ≥40 Years eTable 5. Association Between Annual Ambient Particulate Air Pollution and Bone Mineral Density at the Hip and Lumbar Spine Sites Among Those Aged ≥40 Years eFigure 5. Association Between Biomass Fuel and Hip/Lumbar Spine Bone Mineral Density (BMD) in All Population vs Exposure-Sex Interaction eFigure 6. Association Between Biomass Fuel and Hip/Lumbar Spine Bone Mineral Content Corrected by Bone Area in All Population vs Exposure-Sex Interaction (Among Those Aged ≥40 Years) eFigure 7. Association Between Biomass Fuel and Hip/Lumbar Spine Bone Mineral Density (BMD) in All Population vs Exposure-Sex Interaction (Among Those Aged ≥40 Years) eReferences. [file jamanetwopen-3-e1918504-s001.pdf]

Supplementary Online Content

Ranzani OT, Milà C, Kulkarni B, Kinra S, Tonne C. Association of ambient and household air pollution with bone mineral content among adults in peri-urban South India. *JAMA Netw Open*. 2019;3(1):e1918504. doi:10.1001/jamanetworkopen.2019.18504

eAppendix 1. Missing Data Description and Missingness Pattern
eFigure 1. Directed Acyclic Graph (DAG) Used to Select Confounding Factors
eAppendix 2. Inverse Probability Weighting to Account for Potential Selection Bias
eFigure 2. Associations Between Age and Bone Mineral Content (BMC), Bone Area (BA), and Bone Mineral Density (BMD) by Sex
eFigure 3. Associations Between Bone Mineral Content (BMC) and Bone Area (BA)
eTable 1. Mean Difference (95% CI) in Bone Mineral Content per Interquartile Range Increase in Annual Ambient Particulate Air Pollution, Without Inverse-Probability Correction (IPW)
eFigure 4. Association Between Long-Term Ambient Particulate Air Pollution and Hip/Lumbar Spine Bone Mineral Content Corrected by Bone Area With vs Without Between-Within Exposure Specification
eTable 2. Mean Difference (95% CI) in Bone Mineral Density (BMD) per Interquartile Range Increase in Annual Ambient Particulate Air Pollution
eTable 3. Single and Multi-Pollutant Models for the Mean Difference (95% CI) in Bone Mineral Content and Bone Mineral Density per Interquartile Range Increase in Annual Ambient Particulate Air Pollution
eTable 4. Association Between Annual Ambient Particulate Air Pollution and Bone Mineral Content at the Hip and Lumbar Spine Sites Among Those Aged ≥40 Years
eTable 5. Association Between Annual Ambient Particulate Air Pollution and Bone Mineral Density at the Hip and Lumbar Spine Sites Among Those Aged ≥40 Years
eFigure 5. Association Between Biomass Fuel and Hip/Lumbar Spine Bone Mineral Density (BMD) in All Population vs Exposure-Sex Interaction
eFigure 6. Association Between Biomass Fuel and Hip/Lumbar Spine Bone Mineral Content Corrected by Bone Area in All Population vs Exposure-Sex Interaction (Among Those Aged ≥40 Years)
eFigure 7. Association Between Biomass Fuel and Hip/Lumbar Spine Bone Mineral Density (BMD) in All Population vs Exposure-Sex Interaction (Among Those Aged ≥40 Years)
eReferences.

This supplementary material has been provided by the authors to give readers additional information about their work.

© 2019 Ranzani OT et al. *JAMA Network Open.*

eAppendix 1. Missing Data Description and Missingness Pattern

The missing data descriptions and missingness pattern of 3905 adult participants are shown below.

		N	%
**Complete case**		3717	95%
**Participants with at least 1 missing value**		188	5%
**Missing**		**N missing**	**% missing**
Ambient air pollution	Exposure	130	3.33%
Fuel type	Exposure	52	1.33%
Height	Covariate	3	0.08%
Wb-physical activity	Covariate	3	0.08%
Fruits & vegetables	Covariate	3	0.08%
Calcium	Covariate	3	0.08%
Smoking	Covariate	3	0.08%
Occupation	Covariate	2	0.05%
Education	Covariate	2	0.05%
Sex	Covariate	2	0.05%
% fatmass	Covariate	1	0.03%

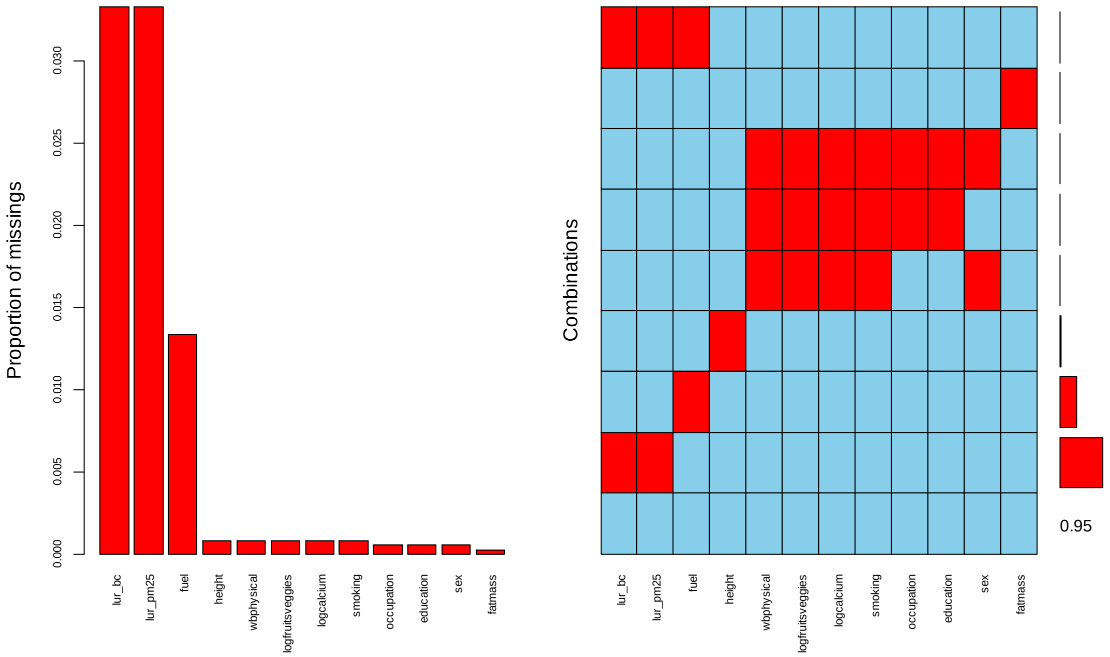

We had a priori decided that we would impute only covariates, but not exposure or outcomes. Without an accurate household geocoding, we believe to multiple impute them would suffer from model misspecification, because the main determinants of ambient air pollution are not in our analytical datasets, such as geographical and land-use indicators. Because there were only 7 participants with missing covariates, we based our analysis on complete case datasets (95%) rather than using multiple imputation.[1] We have clarified this in the revised version. In fact, our target was the adult population 18 years and older: therefore, from 3905 adults, we excluded 181+7 = 188 participants to generate our complete case data, which leads to only 5% of missing values. Given the

© 2019 Ranzani OT et al. *JAMA Network Open.*

relatively small percentage of missing data, it is unlikely that complete case analysis results in materially biased estimates. [1]

© 2019 Ranzani OT et al. *JAMA Network Open.*

eFigure 1. Directed Acyclic Graph (DAG) Used to Select Confounding Factors

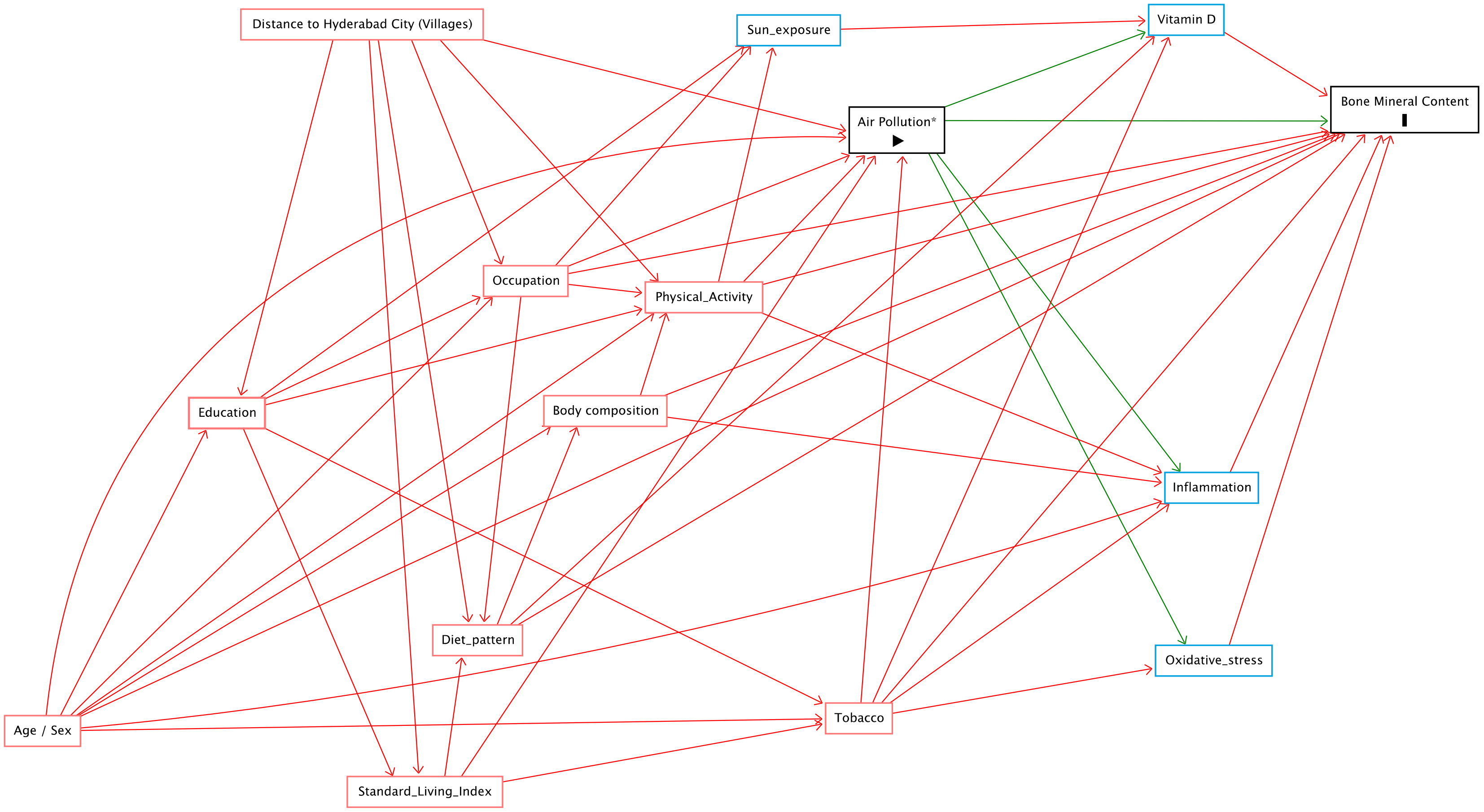

© 2019 Ranzani OT et al. *JAMA Network Open.*

Draw using DAGitty (http://dagitty.net/). Red boxes represent ancestors of the exposure and outcome. Black boxes represent the exposure and outcome. Blue boxes represent outcome ancestors and green lines causal pathways (mediators).[2]

© 2019 Ranzani OT et al. *JAMA Network Open.*

eAppendix 2. Inverse Probability Weighting to Account for Potential Selection Bias

We used inverse probability weighting (IPW)[3] to account for differences between participants included in analyses (N=3717) and the adult population included in APCAPS, representative of the general population. We derived a binary variable describing whether each of the participants was included in the analysis sample (1 if included, 0 if not) and fitted a logistic regression model using the covariates available in the whole cohort (age, sex, villageID, occupation, education, smoking status, weight-bearing physical activity, SLI, height, calcium and fruit and vegetables intake). Being a young male, being unemployed or having an unskilled manual occupation, being an active smoker and eating more fruit and vegetables were associated with a higher odds of being in the analysis sample. There were also geographic differences between villages. The p-value of the Hosmer-Lemeshow test of the model was 0.23. We used this model to predict the probability (p) of each participant to be in the analysis sample and we derived weights as w = 1/p. Weights overlapped among participants in and out the analysis sample; the maximum weight was 12.5. We extracted the derived weights for the analysis population and used them in regression analyses, thus creating a pseudo population representative of the larger population.[3]

© 2019 Ranzani OT et al. *JAMA Network Open.*

eFigure 2. Associations Between Age and Bone Mineral Content (BMC), Bone Area (BA), and Bone Mineral Density (BMD) by Sex

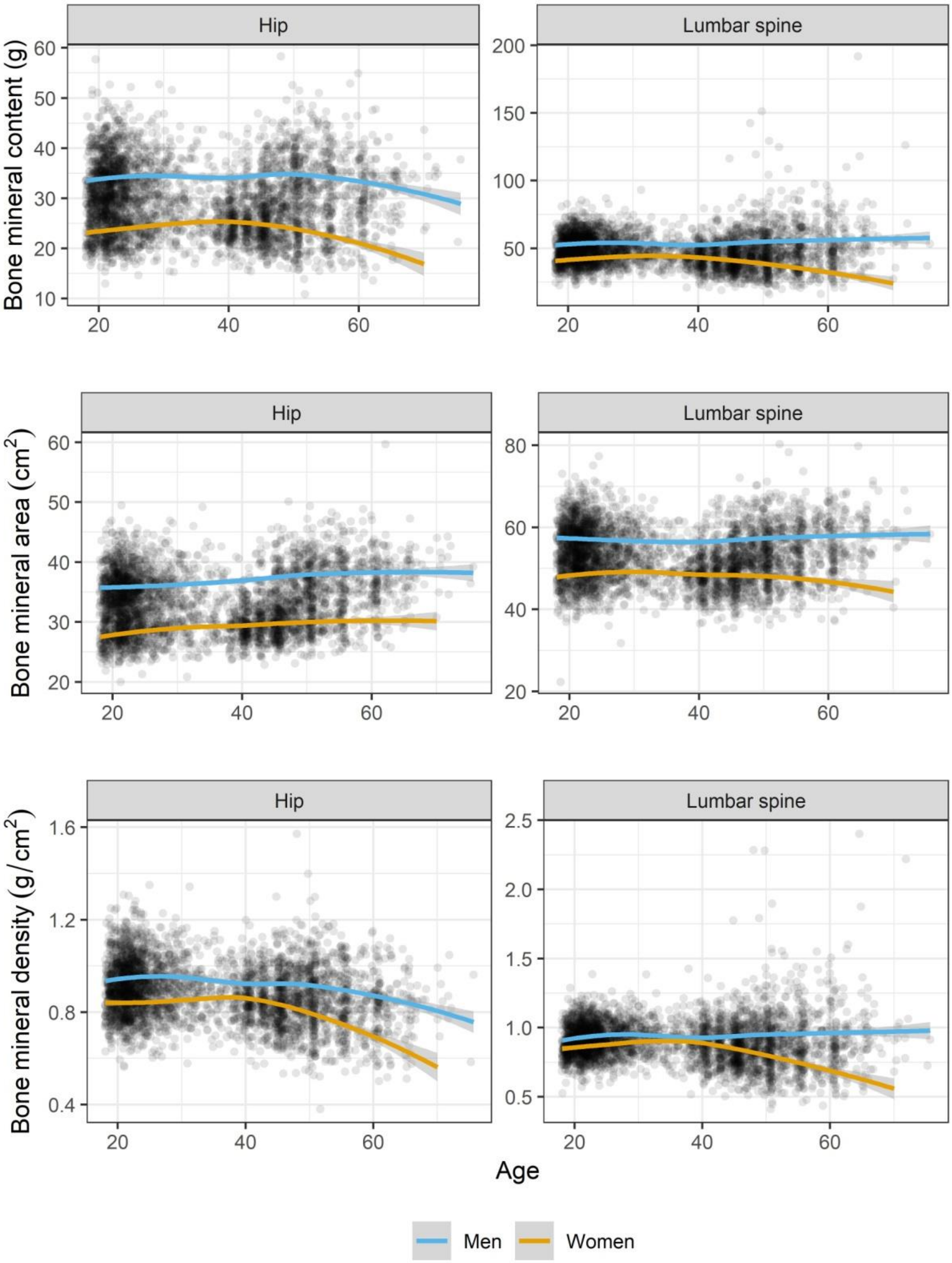

Smooth curves derived using local polynomial regression.

© 2019 Ranzani OT et al. *JAMA Network Open.*

eFigure 3. Associations Between Bone Mineral Content (BMC) and Bone Area (BA)

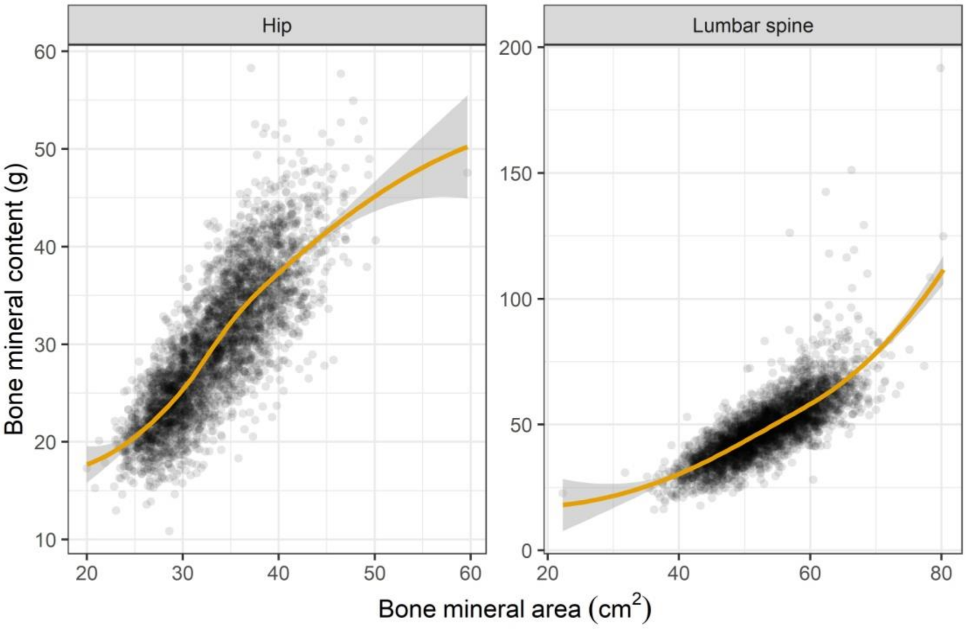

Smooth curves derived using local polynomial regression.

© 2019 Ranzani OT et al. *JAMA Network Open.*

eTable 1. Mean Difference (95% CI) in Bone Mineral Content per Interquartile Range Increase in Annual Ambient Particulate Air Pollution, Without Inverse-Probability Correction (IPW).

	$PM_{2.5}$ per 3 μg/m^3 increase	BC per 1 μg/m^3 increase
Hip (g)	mean difference (95% CI)	mean difference (95% CI)
Model 1[a]	-0.14 (-0.38, 0.1)	-0.76 (-1.54, 0.03)
Model 2[b]	-0.16 (-0.33, 0.01)	-0.37 (-1.01, 0.26)
Model 3[c]	-0.14 (-0.31, 0.02)	-0.34 (-0.97, 0.28)
Model 4[d]	-0.15 (-0.31, 0.02)	-0.34 (-0.96, 0.28)
Lumbar spine (g)		
Model 1[a]	-0.57 (-1.09, -0.06)	-1.04 (-2.76, 0.69)
Model 2[b]	-0.58 (-1.09, -0.07)	-1.01 (-2.71, 0.7)
Model 3[c]	-0.56 (-1.07, -0.06)	-0.99 (-2.68, 0.69)
Model 4[d]	-0.54 (-1.04, -0.04)	-0.97 (-2.64, 0.7)

Mixed effects linear models with nested random intercepts (household within village) without using IPW.

Adjustment:
[a] Model 1: natural spline (bone area) + sex * age + sex * age^2 + DXA machine indicator
[b] Model 2: Model 1 + Height + Fat mass (%) + Lean mass (%)
[c] Model 3: Model 2 + Weight-bearing physical activity + log(Fruit and vegetables intake) + log(Calcium intake) + Current tobacco use + Primary cooking fuel
[d] Model 4: Model 3 + Occupation + Education + Socioeconomic status (SLI index)

BC: black carbon; $PM_{2.5}$: particulate matter less than 2.5 micrometers in aerodynamic diameter

© 2019 Ranzani OT et al. *JAMA Network Open.*

eFigure 4. Association Between Long-Term Ambient Particulate Air Pollution and Hip/Lumbar Spine Bone Mineral Content Corrected by Bone Area With vs Without Between-Within Exposure Specification

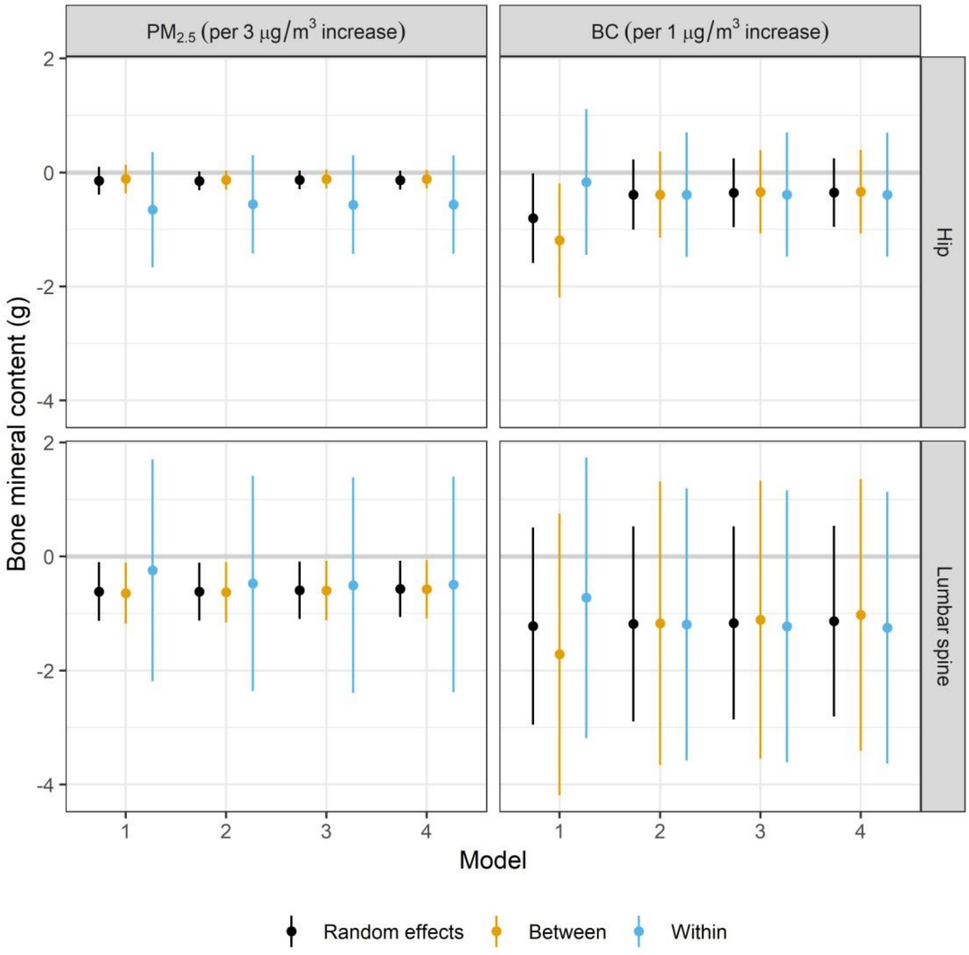

Mixed effects linear models with nested random intercepts (household within village) using inverse-probability weighting (IPW). Two different models with/without between-within specification of the exposure were run.

Adjustment:
Model 1: natural spline (bone area) + sex * age + sex * age^2 + DXA machine indicator
Model 2: Model 1 + Height + Fat mass (%) + Lean mass (%)
Model 3: Model 2 + Weight-bearing physical activity + log(Fruit and vegetables intake) + log(Calcium intake) + Current tobacco use + Primary cooking fuel
Model 4: Model 3 + Occupation + Education + Socioeconomic status (SLI index)
Abbreviations: BC, black carbon; $PM_{2.5}$, particulate matter less than 2 μm in aerodynamic matter.

© 2019 Ranzani OT et al. *JAMA Network Open.*

eTable 2. Mean Difference (95% CI) in Bone Mineral Density (BMD) per Interquartile Range Increase in Annual Ambient Particulate Air Pollution

	$PM_{2.5}$ per 3 µg/m^3 increase	BC per 1 µg/m^3 increase
Hip (g/cm^2)	mean difference (95% CI)	mean difference (95% CI)
Model 1[a]	-0.005 (-0.012, 0.003)	-0.022 (-0.046, 0.002)
Model 2[b]	-0.004 (-0.009, 0.001)	-0.01 (-0.028, 0.008)
Model 3[c]	-0.004 (-0.008, 0.001)	-0.009 (-0.027, 0.009)
Model 4[d]	-0.004 (-0.008, 0.001)	-0.009 (-0.026, 0.009)
Lumbar spine (g/cm^2)		
Model 1[a]	-0.012 (-0.022, -0.002)	-0.019 (-0.052, 0.015)
Model 2[b]	-0.012 (-0.022, -0.001)	-0.013 (-0.046, 0.021)
Model 3[c]	-0.011 (-0.022, -0.001)	-0.013 (-0.046, 0.021)
Model 4[d]	-0.011 (-0.021, 0)	-0.013 (-0.046, 0.021)

Mixed effects linear models with nested random intercepts (household within village) using inverse-probability weighting (IPW). Adjustment:
[a] Model 1: sex * age + sex * age^2 + DXA machine indicator
[b] Model 2: Model 1 + Height + Fat mass (%) + Lean mass (%)
[c] Model 3: Model 2 + Weight-bearing physical activity + log(Fruit and vegetables intake) + log(Calcium intake) + Current tobacco use + Primary cooking fuel
[d] Model 4: Model 3 + Occupation + Education + Socioeconomic status (SLI index)
BC: black carbon; $PM_{2.5}$: particulate matter less than 2.5 micrometers in aerodynamic diameter

© 2019 Ranzani OT et al. *JAMA Network Open.*

eTable 3. Single and Multi-Pollutant Models for the Mean Difference (95% CI) in Bone Mineral Content and Bone Mineral Density per Interquartile Range Increase in Annual Ambient Particulate Air Pollution

	Single-pollutant model	Single-pollutant model	Multi-pollutant model	
	$PM_{2.5}$ per 3 μg/m^3 increase	BC per 1 μg/m^3 increase	$PM_{2.5}$ per 3 μg/m^3 increase	BC per 1 μg/m^3 increase
	mean difference (95% CI)	mean difference (95% CI)	mean difference (95% CI)	mean difference (95% CI)
BMC				
Hip (g)				
Model 4 [a]	-0.13 (-0.3, 0.03), se = 0.083	-0.35 (-0.96, 0.25), se = 0.308	-0.12 (-0.34, 0.09), se = 0.110	-0.07 (-0.85, 0.72), se = 0.401
Lumbar spine (g)				
Model 4 [a]	-0.57 (-1.06, -0.07), se = 0.252	-1.13 (-2.81, 0.54), se = 0.854	-0.56 (-1.18, 0.05), se = 0.314	-0.04 (-2.05, 1.97), se = 1.03
BMD				
Hip (g/cm^2)				
Model 4 [b]	-0.004 (-0.008, 0.001), se =0.002	-0.009 (-0.026, 0.009), se =0.009	-0.004 (-0.010, 0.003), se = 0.003	0 (-0.023, 0.023), se = 0.012
Lumbar spine (g/cm^2)				
Model 4 [b]	-0.011 (-0.021, 0), se =0.005	-0.013 (-0.046, 0.021), se =0.017	-0.013 (-0.025, 0), se = 0.006	0.009 (-0.030, 0.049), se = 0.020

Mixed effects linear models with nested random intercepts (household within village) using inverse-probability weighting (IPW).

Adjustment:
[a] Model 4: natural spline (bone area) + sex * age + sex * age^2 + DXA machine indicator + Height + Fat mass (%) + Lean mass (%) + Weight-bearing physical activity + log(Fruit and vegetables intake) + log(Calcium intake) + Current tobacco use + Primary cooking fuel +Occupation + Education + Socioeconomic status (SLI index)

© 2019 Ranzani OT et al. *JAMA Network Open.*

[b] Model 4: sex * age + sex * age^2 + DXA machine indicator + Height + Fat mass (%) + Lean mass (%) + Weight-bearing physical activity + log(Fruit and vegetables intake) + log(Calcium intake) + Current tobacco use + Primary cooking fuel +Occupation + Education + Socioeconomic status (SLI index)

BC: black carbon; BMC: bone mineral content; BMD: bone mineral density; $PM_{2.5}$: particulate matter less than 2.5 micrometers in aerodynamic diameter; se: coefficient standard error.

© 2019 Ranzani OT et al. *JAMA Network Open.*

eTable 4. Association Between Annual Ambient Particulate Air Pollution and Bone Mineral Content at the Hip and Lumbar Spine Sites Among Those Aged ≥40 Years (n=1639)

	$PM_{2.5}$ per 3 μg/m^3 increase	BC per 1 μg/m^3 increase
Hip (g)	mean difference (95% CI)	mean difference (95% CI)
Model 1[a]	-0.39 (-0.67, -0.12)	-1.56 (-2.49, -0.62)
Model 2[b]	-0.40 (-0.67, -0.13)	-0.69 (-1.68, 0.30)
Model 3[c]	-0.36 (-0.63, -0.10)	-0.63 (-1.60, 0.33)
Model 4[d]	-0.37 (-0.63, -0.11)	-0.69 (-1.64, 0.27)
Lumbar spine (g)		
Model 1[a]	-0.96 (-1.80, -0.11)	-0.92 (-3.78, 1.94)
Model 2[b]	-0.90 (-1.70, -0.09)	-1.17 (-3.91, 1.56)
Model 3[c]	-0.88 (-1.70, -0.06)	-1.17 (-3.93, 1.58)
Model 4[d]	-0.86 (-1.66, -0.06)	-1.20 (-3.91, 1.51)

Mixed effects linear models with nested random intercepts (household within village) using inverse-probability weighting (IPW).

Adjustment:

[a] Model 1: natural spline (bone area) + sex * age + DXA machine indicator

[b] Model 2: Model 1 + Height + Fat mass (%) + Lean mass (%)

[c] Model 3: Model 2 + Weight-bearing physical activity + log(Fruit and vegetables intake) + log(Calcium intake) + Current tobacco use + Primary cooking fuel

[d] Model 4: Model 3 + Occupation + Education + Socioeconomic status (SLI index)

BC: black carbon; $PM_{2.5}$: particulate matter less than 2.5 micrometers in aerodynamic diameter.

© 2019 Ranzani OT et al. *JAMA Network Open.*

eTable 5. Association Between Annual Ambient Particulate Air Pollution and Bone Mineral Density at the Hip and Lumbar Spine Sites Among Those Aged ≥40 Years (n=1639)

	$PM_{2.5}$ per 3 µg/m^3 increase	BC per 1 µg/m^3 increase
Hip (g/cm^2)	mean difference (95% CI)	mean difference (95% CI)
Model 1[a]	-0.011 (-0.019, -0.003)	-0.042 (-0.069, -0.014)
Model 2[b]	-0.010 (-0.017, -0.003)	-0.019 (-0.046, 0.008)
Model 3[c]	-0.009 (-0.016, -0.003)	-0.017 (-0.043, 0.008)
Model 4[d]	-0.010 (-0.016, -0.003)	-0.019 (-0.044, 0.007)
Lumbar spine (g/cm^2)		
Model 1[a]	-0.021 (-0.039,-0.002)	-0.016 (-0.075, 0.042)
Model 2[b]	-0.019 (-0.039, 0)	-0.006 (-0.065, 0.054)
Model 3[c]	-0.019 (-0.038, 0.001)	-0.006 (-0.065, 0.054)
Model 4[d]	-0.018 (-0.038, 0.001)	-0.007 (-0.066, 0.052)

Mixed effects linear models with nested random intercepts (household within village) using inverse-probability weighting (IPW). Adjustment:

[a] Model 1: sex * age + DXA machine indicator

[b] Model 2: Model 1 + Height + Fat mass (%) + Lean mass (%)

[c] Model 3: Model 2 + Weight-bearing physical activity + log(Fruit and vegetables intake) + log(Calcium intake) + Current tobacco use + Primary cooking fuel

[d] Model 4: Model 3 + Occupation + Education + Socioeconomic status (SLI index)
BC: black carbon; $PM_{2.5}$: particulate matter less than 2.5 micrometers in aerodynamic diameter.

© 2019 Ranzani OT et al. *JAMA Network Open.*

eFigure 5. Association Between Biomass Fuel and Hip/Lumbar Spine Bone Mineral Density (BMD) in All Population vs Exposure-Sex Interaction

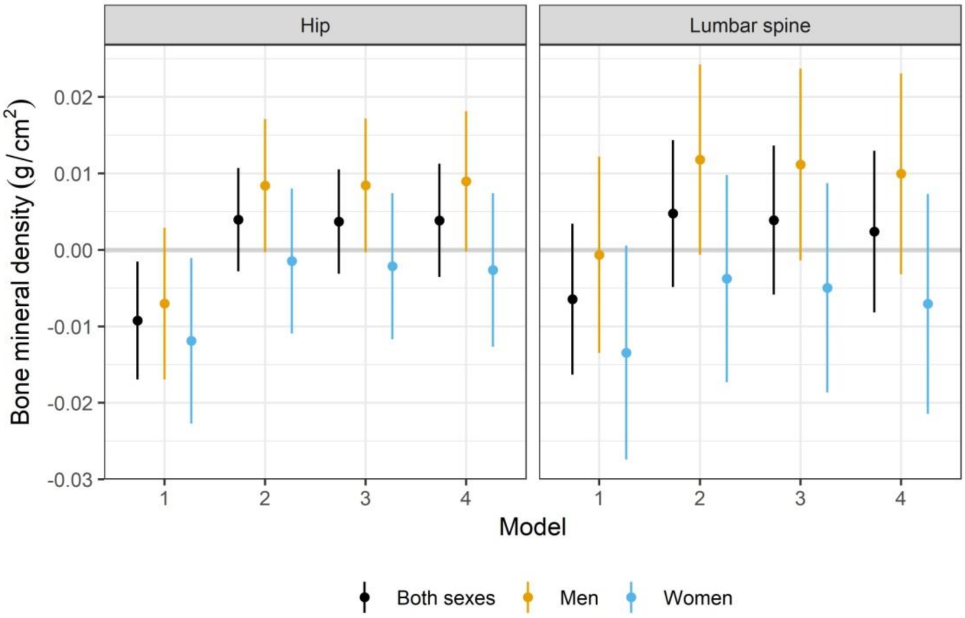

Mixed effects linear models with nested random intercepts (household within village) using IPW. Two different models with/without exposure-sex interactions were run. Adjustment:
Model 1: sex * age + sex * age^2 + DXA machine indicator
Model 2: Model 1 + Height + Fat mass (%) + Lean mass (%)
Model 3: Model 2 + Weight-bearing physical activity + log(Fruit and vegetables intake) + log(Calcium intake) + Current tobacco use
Model 4: Model 3 + Occupation + Education + Socioeconomic status (SLI index)

© 2019 Ranzani OT et al. *JAMA Network Open.*

eFigure 6. Association Between Biomass Fuel and Hip/Lumbar Spine Bone Mineral Content Corrected by Bone Area in All Population vs Exposure-Sex Interaction (Among Those Aged ≥40 Years)

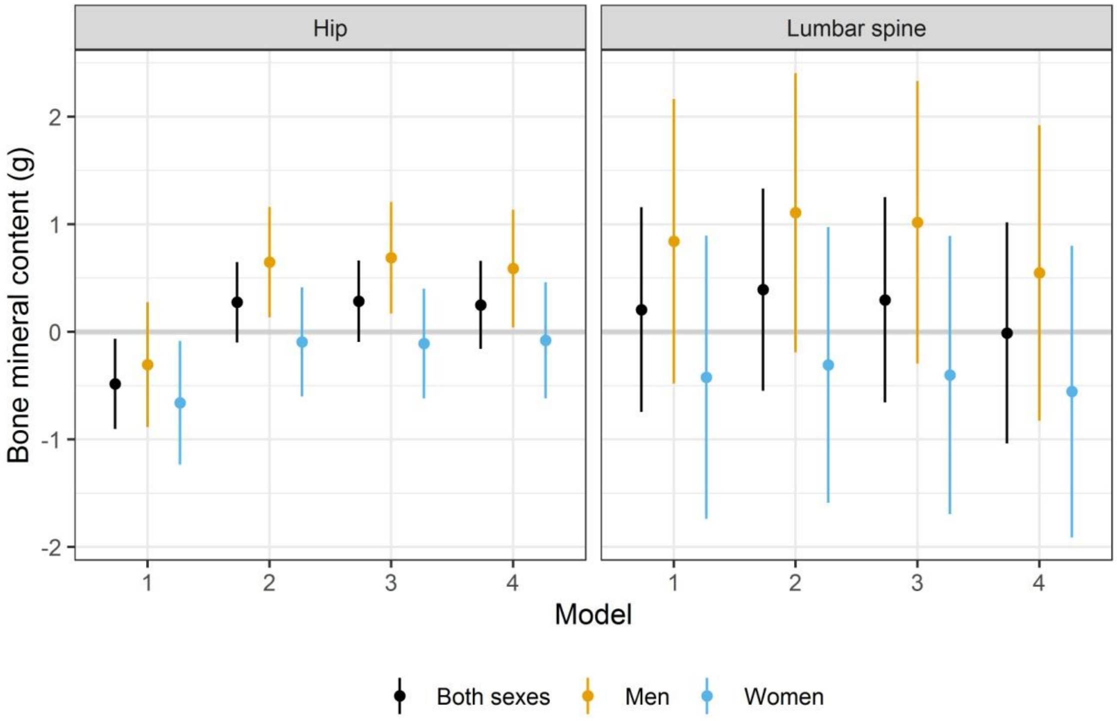

Mixed effects linear models with nested random intercepts (household within village) using inverse-probability weighting (IPW). Two different models with/without exposure-sex interactions were run. Adjustment:

[a] Model 1: natural spline (bone area) + sex * age + DXA machine indicator

[b] Model 2: Model 1 + Height + Fat mass (%) + Lean mass (%)

[c] Model 3: Model 2 + Weight-bearing physical activity + log(Fruit and vegetables intake) + log(Calcium intake) + Current tobacco use

[d] Model 4: Model 3 + Occupation + Education + Socioeconomic status (SLI index)

© 2019 Ranzani OT et al. *JAMA Network Open.*

eFigure 7. Association Between Biomass Fuel and Hip/Lumbar Spine Bone Mineral Density (BMD) in All Population vs Exposure-Sex Interaction (Among Those Aged ≥40 Years)

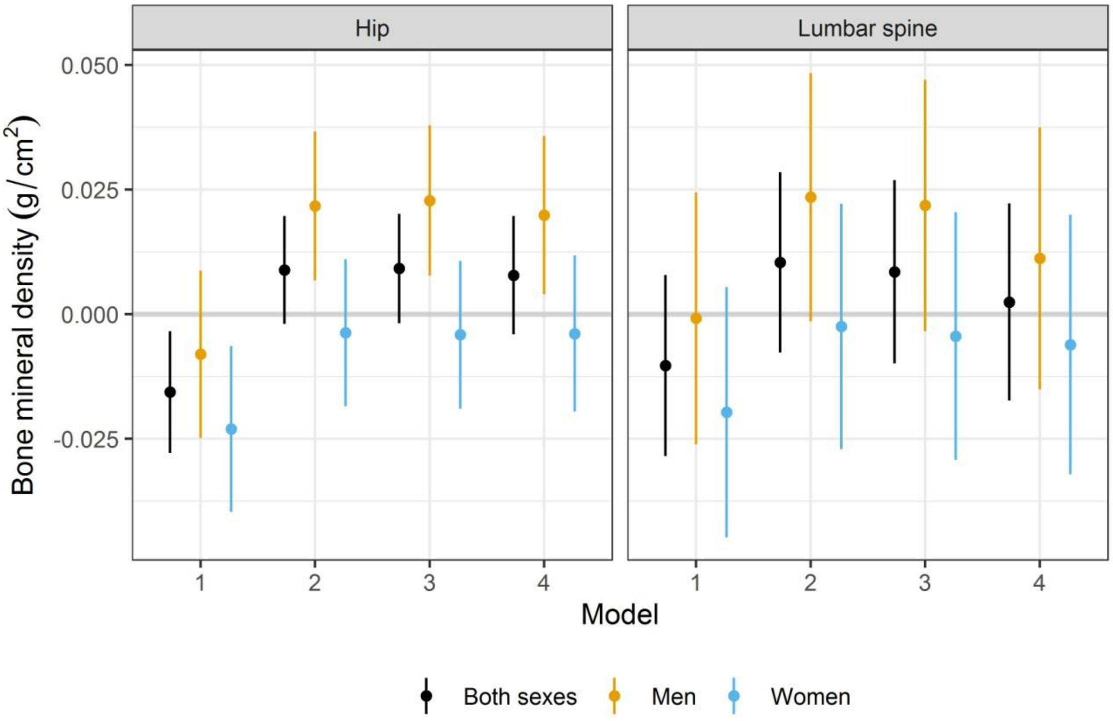

Mixed effects linear models with nested random intercepts (household within village) using IPW. Two different models with/without exposure-sex interactions were run. Adjustment:
Model 1: sex * age + DXA machine indicator
Model 2: Model 1 + Height + Fat mass (%) + Lean mass (%)
Model 3: Model 2 + Weight-bearing physical activity + log(Fruit and vegetables intake) + log(Calcium intake) + Current tobacco use
Model 4: Model 3 + Occupation + Education + Socioeconomic status (SLI index)

© 2019 Ranzani OT et al. *JAMA Network Open.*

eReferences

1. Hughes RA, Heron J, Sterne JAC, Tilling K. Accounting for missing data in statistical analyses: multiple imputation is not always the answer. *Int J Epidemiol*. 2019;48(4):1294-1304. doi:10.1093/ije/dyz032
2. Lederer DJ, Bell SC, Branson RD, et al. Control of confounding and reporting of results in causal inference studies. *Ann Am Thorac Soc*. 2019;16(1):22-28. doi:10.1513/AnnalsATS.201808-564PS
3. Seaman SR, White IR. Review of inverse probability weighting for dealing with missing data. *Stat Methods Med Res*. 2013;22(3):278-295. doi:10.1177/0962280210395740

© 2019 Ranzani OT et al. *JAMA Network Open.*
